# *ABO* genotypes and the risk of esophageal and gastric cancers

**DOI:** 10.1186/s12885-021-08334-1

**Published:** 2021-05-22

**Authors:** Yingxi Chen, Nan Hu, Linda Liao, Kai Yu, Xiao-Ou Shu, Wei Zheng, Jian-Min Yuan, Woon-Puay Koh, You-Lin Qiao, Jin-Hu Fan, Sanford M. Dawsey, Neal D. Freedman, Philip R. Taylor, Alisa M. Goldstein, Christian C. Abnet

**Affiliations:** 1 Division of Cancer Epidemiology and Genetics, National Cancer Institute, National Institutes of Health, 9609 Medical Center Dr. 6E3280, Rockville, MD 20850 USA; 2 Department of Medicine, Division of Epidemiology, Vanderbilt Epidemiology Center, Vanderbilt-Ingram Cancer Center, Vanderbilt University Medical Center, Nashville, TN USA; 3 UPMC Hillman Cancer Center, University of Pittsburgh, Pittsburgh, PA USA; 4 Department of Epidemiology, Graduate School of Public Health, University of Pittsburgh, Pittsburgh, PA USA; 5 Health Service and Systems Research, Duke-NUS Medical School, Singapore, 169857 Singapore; 6 Saw Swee Hock School of Public Health, National University of Singapore, Singapore, 117549 Singapore; 7 National Cancer Center, National Center for Cancer Clinical Research, The Cancer Institute, Chinese Academy of Medical Sciences/Peking Union Medical College, Beijing, China

**Keywords:** ABO blood type, Esophageal squamous cell carcinoma, Gastric cancer

## Abstract

**Background:**

Blood type has been associated with the risk of gastric cancer, but few studies have examined the association with esophageal squamous cell carcinoma (ESCC).

**Methods:**

We conducted a case-control study using genotyping data of Chinese individuals, including cases of 2022 ESCC, 1189 gastric cardia adenocarcinoma, 1161 gastric noncardia adenocarcinoma, and 2696 controls. Genetic blood type was imputed using three single nucleotide polymorphisms. We used logistic regression to examine the association between blood type and the risk of each cancer.

**Results:**

Compared to blood type O, the risk of ESCC was significantly elevated for blood type B and AB, with the highest risk for type AB (OR, 95%CI: 1.34, 1.07–1.67). Analysis of genotype suggested that the association of ESCC was from carrying the *B* allele. Similarly, blood type was significantly associated with gastric noncardia adenocarcinoma (*P* < 0.001) with risk significantly elevated in type A (1.37, 1.14–1.65) and AB (1.44, 1.10–1.89) compared to type O. Blood type was not associated with gastric cardia adenocarcinoma (*P* = 0.13).

**Conclusions:**

This study provides novel insights into the association between blood type and the risk of ESCC and restricted previously observed association to only gastric noncardia cancer, providing important evidence to clarify the pattern of association and suggesting mechanisms of action.

## Background

As early as the 1950s [1], blood type A was reported to be associated with an increased risk of gastric cancer [2]. Advances in genotyping technology have allowed for a more thorough interrogation of the effect of each allele from the ABO locus. A case-control study using genotyping data from a Japanese population found an increased risk of gastric cancer from carrying the *A* allele and a decrease in risk from carrying the *B* allele [3]. Recently, both epidemiologic and genome-wide association studies have demonstrated distinct patterns for gastric cancer when the stomach is divided into the cardia (the most proximal few centimeters of the organ) and the noncardia (the remainder of the stomach) [4–6], suggesting a need for separating gastric cancer by anatomic subsite in etiologic studies.

Despite a long history of research in gastric cancer, few studies have examined the association between ABO blood type and the risk of esophageal cancer [7–9]. One recent case-control study of 480 patients with esophageal squamous cell carcinoma (ESCC) and 480 controls suggested a higher frequency of blood type B in patients with ESCC [10]. However, the study was limited by a relatively small sample size and no replication study to validate the findings.

The aim of the current study was to evaluate the association between ABO blood type and the risk of three upper gastrointestinal cancers—ESCC, gastric cardia adenocarcinoma, and gastric noncardia adenocarcinoma—using genotyping data from five epidemiologic studies of subjects of Chinese ethnicity. Examination of the association between ABO blood type and each of the three upper gastrointestinal cancer sites will provide important evidence to clarify the pattern of association.

## Methods

The study consisted of five distinct study populations of Chinese ethnicity that have been described in detail previously [11–15]. These five studies include a large neighborhood-based case-control study (the Shanxi UGI Cancer Genetics Project); and nested groups from four cohort studies: the Linxian Nutrition Intervention Trial, the Shanghai Men’s Health Study, the Shanghai Women’s Health Study, and the Singapore Chinese Health Study. In the current study, we used GWAS data from the five studies [16], which totaled 2022 ESCC cases, 1189 gastric cardia adenocarcinoma, 1161 gastric noncardia adenocarcinomas, and 2696 control subjects.

### Single nucleotide polymorphism selection and genotyping

The methods of genotyping have been described in detail elsewhere [16, 17]. In summary, genome-wide scanning was performed using the Illumina 660 W array. We excluded SNPs with a missing rate > 5%; subjects with a completion rate of all SNPs < 94%; subjects with abnormal mean heterozygosity values (> 30% or < 25%); gender discordant subjects; or unexpected duplicate pairs [17]. To determine blood type from the SNPs, we first used PHASE [18] to infer haplotypes for rs505922, rs8176746, and rs574347, which provided two *ABO* alleles for each subject [19]. Each subject has two *ABO* alleles contributing to six genotypes, *OO*, *AO*, *AA*, *AB*, *BO*, and *BB*. Phenotypes were determined based on the dominance of the *A* and *B* alleles over the *O* allele. We then used phenotype and genotype data in our subsequent statistical analyses. The fourth SNP that marks the *A2* allele, rs8176704, was nearly monomorphic (MAF < 0.5%) in our study population, and therefore was excluded from analysis.

### Statistical analysis

We used logistic regression models to examine the association between blood type and the risk of the three cancers. All regression results, except the study stratified models, were from models conditioned on study, though we note that this conditioning had little effect on the results in either crude or multivariable adjusted models (data not shown). We adjusted the models for age (in three categories: < 50 years, 50–59 years, and ≥ 60 years), sex, tobacco smoking (never and ever), and alcohol drinking (never and ever). *P*-values were calculated using the difference in − 2 log likelihood of nested models and the chi-square distribution with appropriate degrees of freedom. We used an alpha of 0.05 without correction for multiple comparisons for two reasons: 1) a small number of association tests for each cancer outcome; and 2) a strong prior association for gastric cancers.

To address potential differences in associations by strata across the five study populations, we used a meta-analysis framework to minimize the effect of heterogeneity. We firstly conducted unconditional logistic regressions to estimate the Odds Ratios (ORs) of the blood type and risk of the three cancers by each individual study population. We then used study specific ORs from unconditional logistic regression models to calculate random effects meta-estimates via the *metan* command group in Stata version 11. The remainder of the analysis was conducted using SAS version 9.

## Results

Table 1 presents the characteristics of 2696 control subjects by blood type. Overall, about three-quarters of controls were blood type O (33.0%), follow by blood type B (31.6%) and A (26.9%). Only 8.5% controls were blood type AB.

**Table 1:** Characteristics by imputed ABO blood types^a^ in 2696 control subjects

Characteristic	Blood Type				***P***
	O	A	B	AB	
**N (%)**	889 (33.0%)	725 (26.9%)	853 (31.6%)	229 (8.5%)	
**Age, years**					0.77
< 50, N (%)	193 (21.7%)	174 (24.0%)	200 (23.4%)	48 (21.0%)	
50–59, N (%)	307 (34.5%)	224 (30.9%)	278 (32.6%)	84 (36.7%)	
≥ 60, N (%)	389 (43.8%)	327 (45.1%)	375 (44.0%)	97 (42.2%)	
**Sex**					0.13
Female, N (%)	299 (33.6%)	261 (36.0%)	330 (38.7%)	76 (33.2%)	
Male, N (%)	590 (66.4%)	464 (64.0%)	523 (61.3%)	153 (66.8%)	
**Tobacco smoking**					0.82
Never smoking, N (%)	438 (49.3%)	369 (50.9%)	427 (50.1%)	109 (47.6%)	
Ever smoking, N (%)	451 (50.7%)	356 (49.1%)	426 (49.9%)	120 (52.4%)	
**Alcohol drinking**					0.77
Never drinking, N (%)	745 (83.8%)	595 (82.1%)	701 (82.2%)	189 (82.5%)	
Ever drinking, N (%)	144 (16.2%)	130 (17.9%)	152 (17.8%)	40 (17.5%)	

^a^Blood types imputed from genotype data at rs505922, rs8176746, and rs574347

Table 2 presents the distribution of blood types, stratified by study. We observed evidence for a significant heterogeneity in blood type distributions across studies (*P* = 0.004). Compared with the other four studies, the distribution in the Singapore cohort was distinctly different, with a lower proportion of blood type AB (4.4%) and a higher proportion of blood type O (41.9%).

**Table 2:** Distribution of ABO blood types in control subjects by study

Study	Blood Type				***P***
	O	A	B	AB	
**Overall**	889 (33.0%)	725 (26.9%)	853 (31.6%)	229 (8.49%)	0.004
**Linxian Nutrition Intervention Trial**^**a**^	133 (29.7%)	106 (23.7%)	160 (35.7%)	49 (10.9%)	
**Shanxi Case-Control Study**^**b**^	555 (33.5%)	430 (26.0%)	535 (32.3%)	135 (8.16%)	
**Singapore Chinese Health Study**^**c**^	57 (41.9%)	38 (27.9%)	35 (25.7%)	6 (4.41%)	
**Shanghai Men’s Health Study**^**d**^	73 (34.3%)	67 (31.5%)	56 (26.3%)	17 (7.98%)	
**Shanghai Women’s Health Study**^**e**^	71 (29.1%)	84 (34.4%)	67 (27.5%)	22 (9.02%)	

^a^The Linxian General Population Nutrition Intervention Trial was a large-scale, randomized, double-blind, primary prevention trial in Linxian, China

^b^The Shanxi Case-Control study was a neighborhood matched case-control study conducted in Shanxi, China

^c^The Singapore Chinese Health Study was large-scale cohort study of diet and health in Chinese men and women aged 45–74 years in Singapore

^d^The Shanghai Men’s Health Study was a population-based cohort study of men aged 40–74 years, living in urban Shanghai, China

^e^The Shanghai Women’s Health Study was a population-based cohort study of men aged 40–74 years, living in urban Shanghai, China

Table 3 shows the proportions of blood type and crude and adjusted odds ratios (aOR) for the risk of ESCC, gastric cardia adenocarcinoma, and gastric noncardia adenocarcinoma. Compared to controls, blood type had significantly different distributions for ESCC (*P* = 0.003) and gastric noncardia adenocarcinoma (*P* = 0.001), but not for gastric cardia adenocarcinoma (*P* = 0.39). For ESCC, blood types B (aOR, 95%CI: 1.19, 1.02–1.38) and AB (aOR, 95%CI: 1.34, 1.07–1.67) had significantly increased risk compared to blood type O. For gastric noncardia adenocarcinoma, compared to blood type O, the risk was significantly increased in blood types A (aOR, 95%CI: 1.37, 1.14–1.65) and AB (aOR, 95%CI: 1.44, 1.10–1.89) (Table 3).

**Table 3:** Distribution of imputed ABO blood types^a^ and the risk of upper gastrointestinal cancer

Group	Total	Blood Type			
	N	O	A	B	AB
Number of controls (%)	2696	889 (33.0%)	725 (26.9%)	853 (31.6%)	229 (8.5%)
Number of ESCC cases (%)	2022	581 (28.8%)	527 (26.1%)	701 (34.8%)	208 (10.3%)
Crude^b^ OR (95% CI)		REF	1.18 (1.00–1.38)	1.21 (1.04–1.41)	1.35 (1.08–1.69)
Adjusted^c^ OR (95% CI)		REF	1.17 (1.00–1.38)	1.19 (1.02–1.38)	1.34 (1.07–1.67)
Number of Cardia cases (%)	1189	362 (30.7%)	321 (27.2%)	383 (32.4%)	115 (9.7%)
Crude^b^ OR (95% CI)		REF	1.12 (0.94–1.35)	1.06 (0.89–1.26)	1.19 (0.92–1.55)
Adjusted^c^ OR (95% CI)		REF	1.13 (0.94–1.36)	1.07 (0.89–1.27)	1.17 (0.90–1.53)
Number of Noncardia cases (%)	1161	339 (29.3%)	375 (32.4%)	330 (28.5%)	113 (9.8%)
Crude^b^ OR (95% CI)		REF	1.38 (1.15–1.66)	1.13 (0.94–1.36)	1.47 (1.12–1.91)
Adjusted^c^ OR (95% CI)		REF	1.37 (1.14–1.65)	1.12 (0.93–1.35)	1.44 (1.10–1.89)

^a^Blood types imputed from genotype data at rs505922, rs8176746, and rs574347

^b^Crude logistic models stratified on study

^c^Study stratified models adjusted for age, sex, alcohol consumption, and ever smoking tobacco

To further explore the observed associations, we classified subjects by genotype (Table 4). For ESCC, the association of ESCC was from carrying the *B* allele without allele dose response observed. For noncardia gastric adenocarcinoma, carriage of an *A* allele in any combination was the primary contributor to the increased risk, but again no allele dose response was evident (Table 4).

**Table 4:** Risk^a^ of upper gastrointestinal cancer by genotype

	Allele 2			***P***^**b**^
	***O***	***A***	***B***	
**ESCC**				
**Allele 1**				
***O***	REF	1.17 (0.99–1.39)	1.16 (0.99–1.36)	0.094
***A***	–	1.14 (0.84–1.56)	1.33 (1.06–1.67)	
***B***	–	–	1.31 (1.00–1.72)	
**Cardia**				
**Allele 1**				
***O***	REF	1.15 (0.95–1.40)	1.11 (0.92–1.34)	0.57
***A***	–	1.07 (0.73–1.55)	1.19 (0.91–1.55)	
***B***	–	–	0.93 (0.66–1.30)	
**Noncardia**				
**Allele 1**				
***O***	REF	1.37 (1.13–1.66)	1.11 (0.92–1.35)	0.014
***A***	–	1.35 (0.95–1.92)	1.44 (1.10–1.88)	
***B***	–	–	1.14 (0.81–1.61)	

^a^OR (95% CI) from adjusted logistic model conditioned on study, adjusted for age, sex, alcohol consumption, and ever smoking tobacco

^b^5 df likelihood ratio test

## Discussion

In the current study, we found common and distinct patterns of association between blood type and three upper gastrointestinal cancers in ethnic Chinese populations. Compared to blood type O, blood type AB was associated with the highest risk of ESCC and gastric noncardia adenocarcinoma. Analysis of alleles suggested that the ESCC association was from carrying the *B* allele, with genotypes *AB* and *BB* having the highest risk compared to genotype *OO*. For gastric noncardia adenocarcinoma, the association was from the carriage of one *A* alleles. However, blood type (i.e. phenotype) or allele associations were not observed for gastric cardia adenocarcinoma.

Few studies have examined the association between blood type and the risk of esophageal cancer. Previously, a study of Japanese ESCC patients and controls found a higher frequency of the B antigen among ESCC patients [10]. In the current study, we showed that all non-O blood types had increased risk of ESCC relative to blood type O, but the strongest risk was associated with a carriage of the *B* allele, specifically by genotypes *AB* and *BB*.

The association between blood type and gastric cancer has been widely studied [2, 20]. In this study we confirmed a significantly increased risk of carrying an *A* allele for gastric noncardia adenocarcinoma, regardless of the second allele (*O*, or *B*). Hypothesized mechanisms include physiological differences related to blood type A that mediate the alterations in systemic inflammatory state, intercellular adhesion and membrane signaling, and immune surveillance for malignant cell [21]. In addition, a Chinese case-control study and meta-analysis showed that gastric cancer patients with blood type A had a higher likelihood of *Helicobacter pylori* infections than patients with other blood types, suggesting more frequent infections or greater inflammations in response to infections related to blood type A [22].

Unexpectedly, neither blood type nor allele was associated with gastric cardia adenocarcinoma. This observation is in contrast to previous studies that have shown that in China, gastric cardia adenocarcinoma and ESCC share many risk factors [23] including multiple genetic risk factors [16, 24]. Gastric cardia and noncardia adenocarcinoma have also been observed to share multiple genetic and nongenetic factors that are distinct from the esophagus, for example, *H. pylori* and serum pepsinogens [25, 26]. Neither of these patterns, however, was observed in the current study of blood types, where we found distinct associations with ESCC and gastric noncardia adenocarcinoma but no association with gastric cardia adenocarcinoma. These findings were unlikely to be due to low statistical power for gastric cardia adenocarcinoma, as we had equally large numbers of cases for both gastric cardia (*n* = 1189) and noncardia (*n* = 1161) adenocarcinomas.

Our study has some strengths and limitations. We used an existing dataset that previously identified multiple validated significant associations between ESCC, gastric cardia or noncardia adenocarcinoma and numerous SNPs [16, 17]. A large sample size provided good power overall and the genetic data allowed for an investigation of the underlying genotype, in addition to phenotype. However, a lack of data on *H. pylori* for the full dataset limited insights into this potential disease-associated mechanism, although we note that *H. pylori* infection in our study population is relatively high in controls (92%) [27], leaving little variation in exposure. Moreover, we were not able to conduct re-review of case identification within the study as well as across studies, and therefore were not able to comment on possible misclassification of gastric cardia and noncardia adenocarcinoma in the studies. Additionally, the distribution of blood type in the Singapore cohort was notably different compared with the blood type distribution in the other four populations, though sensitivity analysis excluding the Singapore population did not alter our results (data not shown). Finally, secretor status has been shown to reportedly modify the cancer risk conferred by ABO blood type [28, 29]. In this study we did not have genotype information on the rare alleles that may confer negative secretor status in people of Chinese descent [30]. We did, however, conduct a sensitivity analysis extracting all available SNPs (*N* = 11) in the *FUT2* gene, a functional *FUT2* allele being required for secretion, from our GWAS data to test whether these singly or collectively altered the association with ABO SNPs. They did not alter our results (data not shown).

## Conclusion

In conclusion, our results validate a previously reported association between blood type and the risk of gastric adenocarcinoma and extended this observation to show that the association was restricted to only gastric noncardia adenocarcinoma. In addition, we found a significantly increased risk for ESCC conveyed by the carriage of the *B* allele. Functional investigations are warranted to elucidate the mechanisms of ABO blood groups behind these associations.

## Data Availability

The datasets used and/or analyzed during the current study are available from the corresponding author on reasonable request.

## Abbreviations

ESCC: Esophageal squamous cell carcinoma
OR: Odds ratio
